# FACTORS ASSOCIATED WITH THE INCIDENCE OF COGNITIVE IMPAIRMENT IN OLDER COSTA RICAN ADULTS

**DOI:** 10.1093/geroni/igad104.3343

**Published:** 2023-12-21

**Authors:** Ericka Méndez-Chacón, David Camacho

**Affiliations:** Universidad de Costa Rica, San Jose, San Jose, Costa Rica; University of Illinois, Chicago, Chicago, Illinois, United States

## Abstract

Cognitive impairment is a critical global public health problem that contributes to lower quality of life and greater medical costs. Despite the growing number of older adults in Costa Rica, few studies have examined cognitive impairment in this population. We drew data on participants (60 years+) with no cognitive impairment (Score of >=12 on MMSE) from wave 1 of the Costa Rican Longevity and Healthy Aging Study (CRELES; n=2,827). Guided by the diathesis-stress-model, we used logistic regression to determine social and health predictors of cognitive impairment incidence (Score < 12 of on MMSE) 2 years later (wave 2). Incidence of cognitive impairment was 5.8% (95% CI, 5.0-7.0). Formal school education (elementary: OR=0.32; 95% CI, 0.17-0.60; high school or more: OR=0.11; 95% CI, 0.02-0.60) and being married or living as married (OR=0.52; 95% CI, 0.28-0.96) were identified as protective against cognitive impairment. Greater age (OR=1.09; 95% CI, 1.06-1.13), consuming more than 400g of carbohydrates per day (OR=2.20; 95% CI, 1.22-3.91), and more challenges with Instrumental Activities of Daily Living (OR=1.70; 95% CI, 1.23-2.28) increased odds of developing cognitive impairment. Common predictors including biological sex, residential zone, smoking, income, bad health perception, depression, and cortisol (ug/dl) were not associated with the onset of cognitive impairment at wave 2. We consider that contextual (e.g., healthcare infrastructure), cultural factors (e.g., familism), as well as diet may help shape predictors and their effects on cognitive functioning. Researchers and practitioners should collaborate on implementation of culturally appropriate and contextually feasible interventions that address predictors of cognitive impairment.

